# The Skin–Brain Connection Hypothesis, Bringing Together CCL27-Mediated T-Cell Activation in the Skin and Neural Cell Damage in the Adult Brain

**DOI:** 10.3389/fimmu.2016.00683

**Published:** 2017-01-16

**Authors:** Nataliya L. Blatt, Timur I. Khaiboullin, Vincent C. Lombardi, Albert A. Rizvanov, Svetlana F. Khaiboullina

**Affiliations:** ^1^Institute of Fundamental Medicine and Biology, Kazan Federal University, Kazan, Russia; ^2^Republican Clinical Neurological Center, Kazan, Russia; ^3^Nevada Center for Biomedical Research, Reno, NV, USA

**Keywords:** melatonin, multiple sclerosis, CCL27, inflammation, brain, skin, light

## Abstract

Recent discovery of an association of low serum melatonin levels with relapse in multiple sclerosis (MS) opens a new horizon in understanding the pathogenesis of this disease. Skin is the main organ for sensing seasonal changes in duration of sunlight exposure. Level of melatonin production is dependent on light exposure. The molecular mechanisms connecting peripheral (skin) sensing of the light exposure and developing brain inflammation (MS) have not been investigated. We hypothesize that there is a connection between the reaction of skin to seasonal changes in sunlight exposure and the risk of MS and that seasonal changes in light exposure cause peripheral (skin) inflammation, the production of cytokines, and the subsequent inflammation of the brain. In skin of genetically predisposed individuals, cytokines attract memory cutaneous lymphocyte-associated antigen (CLA+) T lymphocytes, which then maintain local inflammation. Once inflammation is resolved, CLA+ lymphocytes return to the circulation, some of which eventually migrate to the brain. Once in the brain these lymphocytes may initiate an inflammatory response. Our observation of increased CC chemokine ligand 27 (CCL27) in MS sera supports the involvement of skin in the pathogenesis of MS. Further, the importance of our data is that CCL27 is a chemokine released by activated keratinocytes, which is upregulated in inflamed skin. We propose that high serum levels of CCL27 in MS are the result of skin inflammation due to exposure to seasonal changes in the sunlight. Future studies will determine whether CCL27 serum level correlates with seasonal changes in sunlight exposure, MS exacerbation, and skin inflammation.

## CCL27 Expression

CC chemokine ligand 27 (CCL27) was first described by Ishikawa-Mochizuki et al. as a CC chemokine, named based on the presence of two adjacent cysteines, selectively expressed in skin infected with molluscum contagiosum (1). Subsequently, Morales et al. confirmed the exclusive expression of CCL27 in skin keratinocytes (2). However, later studies demonstrated that CCL27 expression was not restricted to keratinocytes as it was found in normal mucosa-associated colon epithelium, trachea, and mammary glands (3). In addition, CCL27 transcripts were detected in the retinal layer of the eye under normal and inflammatory conditions (4). The fact that retinal cells have a neuronal origin prompted investigation of CCL27 expression in cells within the central nervous system (CNS). Although brain tissue lacks full-length CCL27 transcripts, two alternatively spliced forms of RNA were identified. One form, termed PESKY, is a non-secreted form targeting the nucleus where it modulates transcriptional activity and cytoskeletal actin rearrangement (5). The second form of CCL27 appears to be a truncated peptide of 67 amino acids, which is abundantly expressed in mouse brain tissue (6). Both the truncated RNA and the expressed protein were found predominantly in the olfactory bulbs, dentate gyrus of the hippocampus, amygdala, and the external layer of the cerebellum. In another study conducted by Arimitsu et al., expression of CCL27 was found in freshly isolated human neurons and astrocytes (7). Therefore, these data support the expression of CCL27 beyond skin tissue, including in mucosal epithelium and brain.

Although constitutively present, CCL27 expression can be augmented by exposure to pro-inflammatory stimuli. For example, CCL27 was upregulated in keratinocytes by tumor necrosis factor (TNF)-α and interleukin (IL)-1β (8). In addition, TNF-α activation of CCL27 can be potentiated in the presence of CCL17, a chemokine shown to play a role in induction of mucosal inflammation (9, 10). Therefore, it was suggested that CCL27 may play a role in the pathogenesis of inflammation. This hypothesis has been confirmed by numerous studies where increased serum levels of CCL27 were found in inflammatory conditions such as atopic dermatitis (11), grafts-vs-host disease (12), and psoriasis (13). Reiss et al. have shown that CCL27 promotes inflammation by regulating antigen-induced lymphocyte tissue recruitment (14). In addition, Homey et al. confirmed a specific role of CCL27 in T lymphocyte trafficking into inflamed skin (15). The nature of lymphocytes recruited by CCL27 was investigated by Morales et al. (2). These authors demonstrated that CCL27 selectively recruits cutaneous lymphocyte-associated (CLA^+^) memory T lymphocytes into the skin, while failing to attract CD4^+^, CD8^+^ naive T cells, CD8^+^ memory lymphocytes, B cells, monocytes, or neutrophils. These data suggest that attraction of CLA+ lymphocytes plays a role in the pathogenesis of atopic dermatitis, since lymphocyte accumulation correlates with disease severity.

Interestingly, the role of CCL27 truncated form in allergic brain inflammation was shown by Gunsolly et al (6). Authors have shown upregulation of truncated CCL27 in the cerebral cortex and limbic structures. The transcriptional activation of CCL27 variant 1 (PESKY) was in response to the peripheral allergic inflammation and paralleled the upregulation of T helper 2 (Th2) cytokines IL-4, IL-5, and IL-13. It appears that Th2 stimuli are essential for transcriptional regulation of CCL27 family cytokines. For example, the complete isoform of CCL27 (CTACK) was shown upregulated in allergic skin reaction (16, 17), while transcription of truncated cytokine PESKY increased in brain during olfactory bulb allergic inflammation (6). Since PESKY is exclusively expressed in the CNS, its role in allergic brain inflammation could be suggested.

## CCL27 Function

Published reports suggest that CCL27 has a broader function than just regulation of lymphocyte trafficking. For example, Kraynyak et al. have shown that CCL27 has adjuvant activity, enhancing immune responses to HIV-1 and SIV antigens (18). Animals immunized with HIV-1gag/CCL27 plasmid demonstrated an enhanced immune response at mucosal sites, which was accompanied by high levels of antigen-specific IgA in bronchoalveolar lavage and fecal samples. In addition, increased CD4 counts significantly increased interferon-γ secretion and CD8+ T-cell proliferation in peripheral blood of immunized animals. These data suggested that CCL27 modulation of the immune response is associated with promoting T helper 1 (Th1)-activating antigen-presenting cells. Supporting this assumption data, published by He et al. (19), demonstrated early upregulation of CCL27 in antigen primed IL-10 knockout dendritic cells (DCs). These authors hypothesized that the upregulation of CCL27 by DC was associated with an increased expression of co-stimulatory molecules and activation of Th1 lymphocytes. Therefore, this suggests that the role of the CCL27 chemokine in inflammation involves lymphocyte recruitment and promotion of the Th1 type immune response.

## CCL27 Receptors

CCL27 is a ligand for two CC chemokine receptors (CCR), CCR4 and CCR10. CCR10 is expressed on DCs, memory T lymphocytes, and IgA-secreting mucosal plasma cells (15, 20, 21), while CCR4 is expressed by activated lymphocytes (22). Interestingly, both receptors are known to be major regulators of lymphocyte homing to inflamed skin (15, 23). However, expression of CCR4 and CCR10 are not limited to skin-targeted leukocytes as they have been found to be expressed in astrocytes, the major component of neuroglia (24, 25). Interestingly, Lui et al. demonstrated that the expression of CCR10 is mainly localized to the hippocampus (26), where Gunsolly et al. detected the receptor ligand, CCL27 (6). This suggests that the interaction between CCL27 and its receptors is not exclusive to the skin, but it plays a role in maintenance of the brain homeostasis as well as CNS immune surveillance. In addition, CCL27, released by damaged or activated neurons and astrocytes within the brain, could be a trigger for the chemotaxis of memory T lymphocytes primed in the skin.

## CCL27 Can be Secreted by Astrocytes, a Structural Component of the Blood–Brain Barrier (BBB)

Although the pathogenesis of immune reactivity in neuroinflammatory disease remains largely unknown, leukocyte infiltration is often a hallmark of the disease. For example, leukocytes crossing the BBB was shown at the early stages of multiple sclerosis (MS) (27), a chronic inflammatory disease of the CNS. Therefore, it is generally accepted that the integrity of the BBB is essential for regulation of leukocyte trafficking and establishing CNS inflammation. Astrocytes are major component of the BBB, maintaining permeability and regulating leukocyte trafficking upon activation (28). Interestingly, astrocytes can secrete CCL27, which when released to the nearby BBB, can contribute to leukocyte trafficking (29). However, the BBB may not be the only entry point for leukocytes, as it has been shown by Gunsolly et al. (6), using an animal model, that upregulation of CCL27 in olfactory bulbs after the intranasal allergen challenge was associated with the presence of mature T cells. Thus, it may be hypothesized that T lymphocytes may access the CNS *via* the nasal mucosa, the cribriform plate, and the perineural spaces of the olfactory bulb, bypassing the leukocyte traffic control by BBB (30).

## Memory CLA+ T Cells in MS

Although leukocyte infiltration of brain tissue in MS is well documented, our knowledge of the mechanisms controlling leukocyte trafficking is limited. Studies have shown a role for integrins and selectins in leukocyte recruitment into the CNS, where P-selectin blockade or treatment with anti-α4 integrin antibody partially decreases lymphocyte trans-BBB migration and reduces the severity of experimental autoimmune encephalitis, an animal model of MS (31–33). However, the most interesting observation was that CLA+ T cells were found in the cerebrospinal fluid (CSF) of healthy individuals (34). Expression of CLA antigen is the characteristic for cutaneous lymphocytes, while expression of integrins is indicative of gut homing (35, 36). These findings suggest that lymphocyte homing to the brain involves the recruitment of memory T cells primed outside the CNS. Therefore, immune response to pathogens in skin and gut tissues may influence the intrathecal immune response.

**Figure 1:**
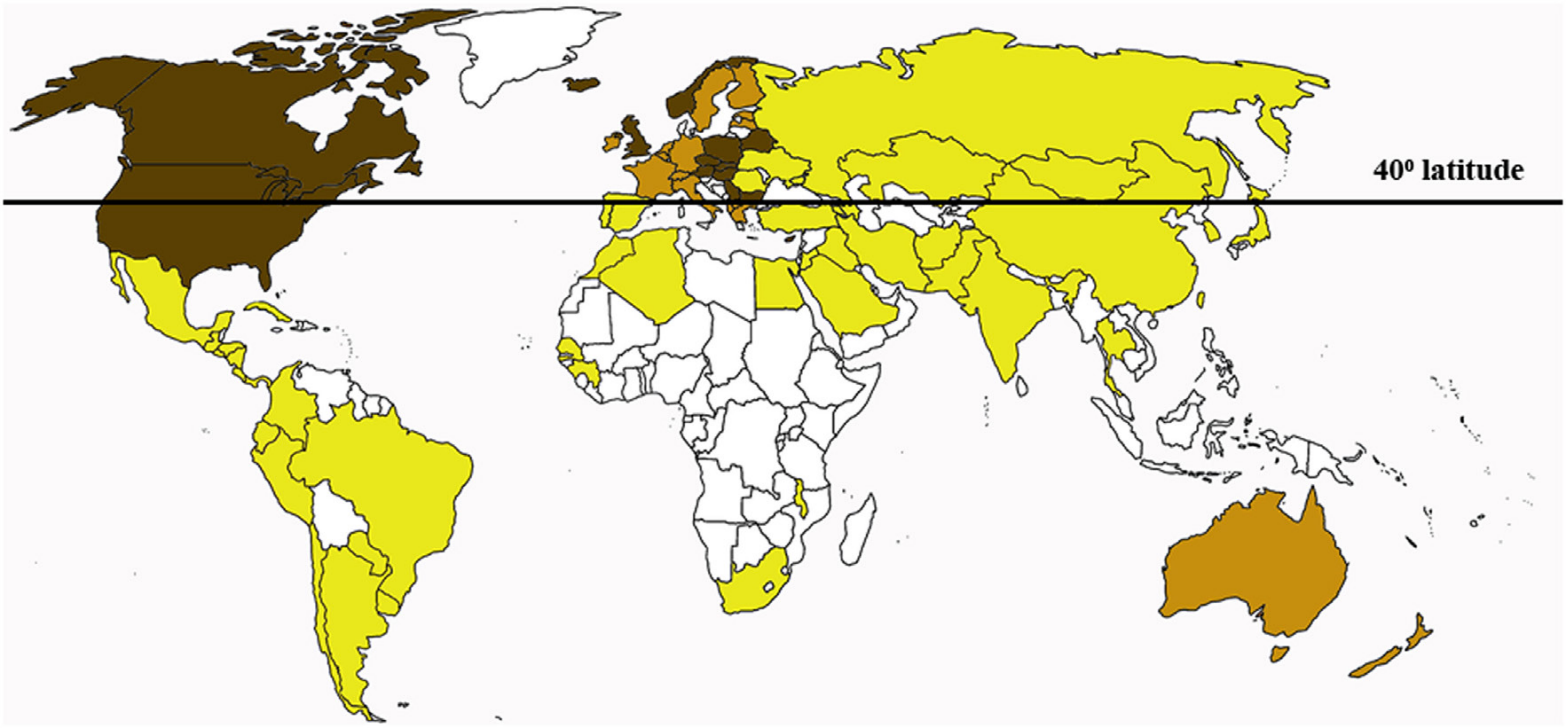
**The multiple sclerosis (MS) risk rate distribution worldwide**. More MS cases are registered in countries with tempered climate north from the 40° latitude. Dark brown, high risk; brown, potentially high risk; yellow, low risk; white, data not available.

Skin is the largest organ providing the first-line defense in infection and injury. Interestingly, the connection between skin sun exposure and the risk of developing MS has been documented. For example, the majority of MS patients reside in temperate regions where sunlight is rarely intense (37, 38) (Figure 1). Even within the same country in northern latitudes, the highest prevalence of MS was found in the northern regions as compared to the south (39, 40). The role of the sunlight exposure in MS pathogenesis is also supported by documented higher frequency of the disease relapse in seasons with higher skin sun exposure and increased solar radiation. For example, Salvi et al. reported increased frequency of MS relapse in May to June as compared to September (41). Also, Meier et al. have shown a likelihood of higher MS activity in March to August as compared to the rest of the year, which was correlated with the changes in solar radiation (42). It has been suggested that both skin color and ultraviolet (UV) exposure play a role in the onset of MS. An increased latitudinal gradient of MS prevalence is documented, where higher incidence rate is registered among patients residing above the 42° latitude (37, 43). Therefore, it was not surprising that the highest prevalence of MS in the world was registered in Scotland and England (44, 45). Indigenous population of these northern European regions developed adaptive changes including maximum skin depigmentation (46). Decreased skin pigmentation promotes vitamin D synthesis, which is especially important in the high latitude where the low UVB rate is characteristic (47). However, the depigmented skin will also have less protection against harmful effect of the damaging sun UV spectrum, thus producing local skin inflammation (48, 49).

Skin color is determined by the type of melanin produced by melanocytes. Dark pigment, eumelanin, protects skin cells from UV damage (50). In contrast, the yellow-red-colored pheomelanin is less effective in providing UV protection (51). Recently, Mitra et al have shown that pheomelanin may even promote oxidative DNA injury by generating free radicals in the absence of UV in fair skin individual (52). Therefore, this suggests that increased oxidation and DNA damage caused by UV exposure in persons with pheomelanin can trigger a cutaneous immune response. The skin immune response is mainly associated with a subset of resident CLA+ memory T cells (35, 53, 54). However, little is known about the dynamics of CLA+ T lymphocytes in MS skin during remission and exacerbation, as well as about the correlation between the type of cutaneous melatonin and CLA+ lymphocytes.

**Figure 2:**
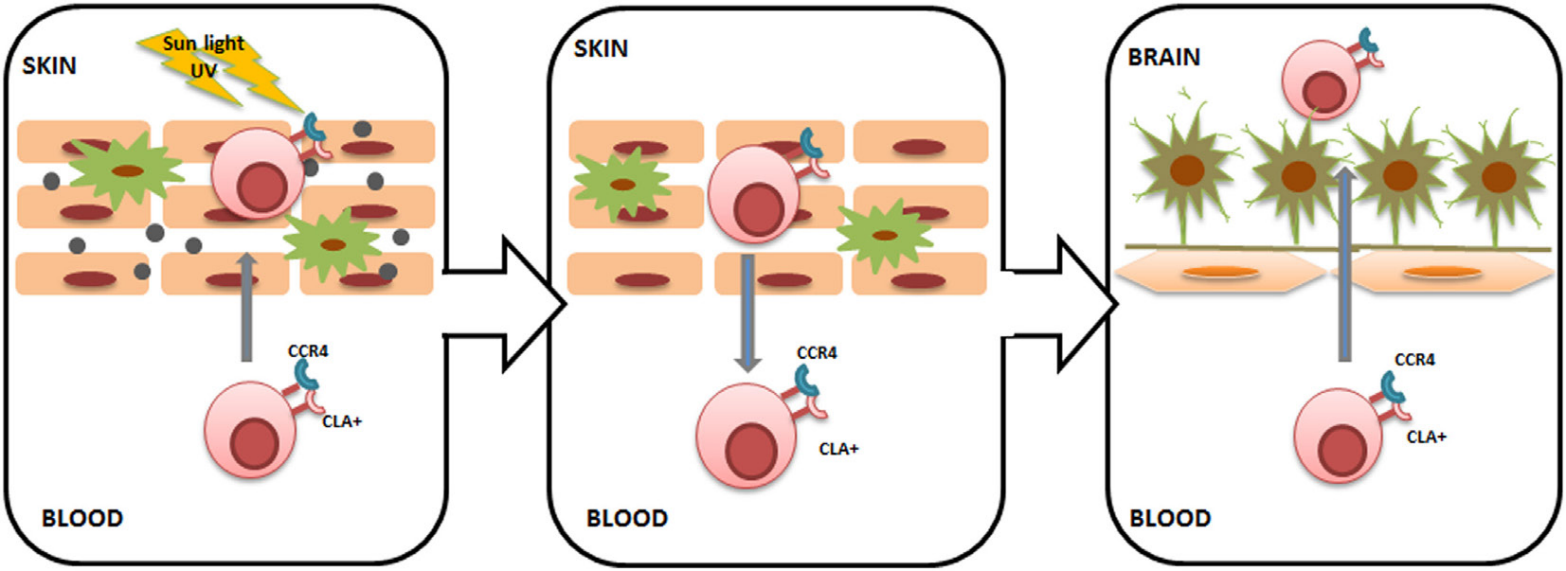
**Cutaneous lymphocyte-associated antigen (CLA)+ T lymphocyte trafficking from the skin to the brain**. Sunlight ultraviolet damage to the skin causes keratinocyte activation and CC chemokine ligand 27 (CCL27) release. CCL27 recruits CLA+/CC chemokine receptor 4 (CCR4)+ T cells into the skin, where they become primed to various cutaneous pathogens as well as antigens and autoantigens. Once skin inflammation is resolved, CLA+/CCR4+ T lymphocytes return back into the circulation and can enter other tissues, including the brain. Within the brain, primed CLA+/CCR4+ T lymphocytes can target brain tissue, triggering inflammation. Long-lasting inflammation within the central nervous system can be ensured by yearly seasonal trans-blood–brain barrier (BBB) migration of activated CLA+/CCR4+ T lymphocytes.

We have previously shown that serum levels of CCL27 are upregulated in subjects with MS (55). CCL27 was two times higher in the serum of acute MS subjects and remained upregulated in the later stages of the disease. The origin of CCL27 in MS serum remains unknown; however, it could be suggested that it is produced in the periphery, for example, in the skin. Increased CCL27 within the skin regulates CLA+ T lymphocyte cutaneous trafficking; therefore, high serum levels of CCL27 are commonly found in inflammatory skin diseases (10, 14). Interestingly, an increased number of circulating CLA+ lymphocytes were also found in inflammatory skin diseases; however, the most striking observation was that circulating CLA+ T cells remained upregulated even during remission (56). As a result, skin-activated CLA+ T cells will recirculate to the blood and will be retained in circulation (57). These recirculating CLA+ T cells target various cutaneous pathogens as well as antigens and autoantigens, as the lymphocyte phenotype will be influenced by the skin environment to which they were exposed. Once in circulation, CLA+ lymphocytes may migrate into other tissues, including the brain (Figure 2). Interestingly, the presence of CLA+ lymphocytes in the CSF of healthy individuals was described by Kivisäkk et al. (34), suggesting that activated memory cells generated in the skin are trafficking into the brain under normal conditions. Therefore, it could be postulated that when the number of circulating cutaneous memory leukocytes increases, more skin-activated lymphocytes will be migrating into the brain. In addition to the CLA marker, cutaneous lymphocytes express CCR4, which was found on a high number of lymphocytes in the CSF in MS (34). These data corroborate the notion that lymphocytes infiltrating CNS in MS may have a cutaneous origin. Furthermore, CCL27 could act as a chemoattractant facilitating intrathecal migration of skin-activated lymphocytes, since this cytokine is a ligand for CCR4 (17).

In conclusion, our observation of increased CCL27 in serum of MS cases suggests a role for this cytokine in pathogenesis of the disease. Although the mechanisms of the contribution of CCL27 in MS pathogenesis remain largely unknown, the fact that CCL27 is a known chemoattractant for skin-derived memory T lymphocytes suggests a connection between cutaneous inflammation and developing MS. We propose that skin damage due to UV exposure and the type of melanin produced by melanocytes may play a role in cutaneous inflammation and development of activated memory T lymphocytes. Once inflammation is resolved, activated skin T lymphocytes are recirculated and become available to migrate into the CNS. After entering the brain *via* many routes, bypassing the BBB, cutaneous T cells may reach brain tissue where lymphocytes could attack neural cells and induce inflammation.

## Author Contributions

NB: writing the manuscript, literature analysis, and creating figures. TK: discussion and intellectual contribution into the clinical aspects of MS. VL: intellectual contribution, discussion, and English editing. SK: instigating the main scope of the review and intellectual contribution in discussion of the review progress with team of authors. AR: organizing the team of authors, providing financial and logistic support, and intellectual contribution in the review outlines.

## Conflict of Interest Statement

The authors declare that the research was conducted in the absence of any commercial or financial relationships that could be construed as a potential conflict of interest.
